# Food Proteins and Bioactive Peptides: New and Novel Sources, Characterisation Strategies and Applications

**DOI:** 10.3390/foods7030038

**Published:** 2018-03-14

**Authors:** Maria Hayes

**Affiliations:** The Food BioSciences Department, Teagasc Food Research Centre, Ashtown, Dublin 15, Ireland; Maria.Hayes@teagasc.ie; Tel.: +353-1-8059957

**Keywords:** protein, bioactive peptides, Protein Digestibility Amino Acid Score (PDCAAS), Digestible Indispensable Amino Acid Score (DIAAS), functional activities, heart health, diabetes, mental health

## Abstract

By 2050, the world population is estimated to reach 9.6 billion, and this growth continues to require more food, particularly proteins. Moreover, the Westernisation of society has led to consumer demand for protein products that taste good and are convenient to consume, but additionally have nutritional and health maintenance and well-being benefits. Proteins provide energy, but additionally have a wide range of functions from enzymatic activities in the body to bioactivities including those associated with heart health, diabetes-type 2-prevention and mental health maintenance; stress relief as well as a plethora of other health beneficial attributes. Furthermore, proteins play an important role in food manufacture and often provide the binding, water- or oil-holding, emulsifying, foaming or other functional attributes required to ensure optimum sensory and taste benefits for the consumer. The purpose of this issue is to highlight current and new protein sources and their associated functional, nutritional and health benefits as well as best practices for quantifying proteins and bioactive peptides in both a laboratory and industry setting. The bioaccessibility, bioavailability and bioactivities of proteins from dairy, cereal and novel sources including seaweeds and insect protein and how they are measured and the relevance of protein quality measurement methods including the Protein Digestibility Amino Acid Score (PDCAAS) and Digestible Indispensable Amino Acid Score (DIAAS) are highlighted. In addition, predicted future protein consumption trends and new markets for protein and peptide products are discussed.

The increasing global population and the need for reduction of or alternatives to animal protein consumption are two factors currently driving the development of new and alternative protein research and product development along with increased consumer awareness of the need to reduce carbon emissions. In addition, it has been identified that plant-based proteins from the likes of lentils, fava beans, peas and dry beans and soy are beneficial for health including the cardiovascular system, in glycaemic control, for the provision of minerals, vitamins, and fibre. 

In this edition, Maehre and colleagues review the best method for use in the determination of protein quantities and qualities in food products and ingredients and conclude that the amino acid analysis method is the only one currently available where interference doesn’t occur from some other substance, such as carbohydrates, lectins and anti-nutritional factors [1]. Inaccuracies were linked to indirect measurements, i.e., nitrogen determination and subsequent conversion to protein. In addition, novel protein processing methods were reviewed and implemented on soy protein. Vagadia and colleagues looked at conventional and microwave treatment methods for the inactivation of Trypsin inhibitors in order to improve in vitro digestibility of this plant protein source. Both microwave and conventional treatments were found to increase the digestibility of soymilk by 7 and 11%, respectively, and also reduced Trypsin inhibitor activities by 1 and 3%, respectively. This finding is important as, if implemented commercially it could increase the nutritional quality of soymilk by removal of anti-nutritional factors thereby improving the nutritional health of consumer groups including vegans and vegetarians as well as those that choose soymilk instead of traditional cow’s milk due to lactose intolerance [2].

The generation of protein hydrolysates and ingredients is a strategy used by industry to improve the solubility of proteins. In addition to this functional benefit, hydrolysis is a strategy that may be employed to generate health benefits to protein ingredients as it results in the creation of bioactive peptides that often have hormone-like beneficial health effects once consumed. Bioactive peptides can be generated from any protein source using hydrolysis with commercially available enzymes or fermentation with proteolytic lactic acid bacteria (LAB). Importantly, they are inactive within the parent protein sequence and must be released before becoming active. In addition, they must survive the conditions within the gastrointestinal tract and reach their targets intact and in sufficient concentrations to provide an in vivo, beneficial health effect [3,4]. Daliri et al. [5] provide a detailed overview of the potential health benefits of bioactive peptides as well as a number of the challenges that exist concerning their development. Albenzio reviewed the use of bioactive peptides in animal food products [6]. Bleakley and colleagues look at the potential health benefits of cereal proteins using a novel strategy of combining an in silico computer-based screening approach with traditional protein extraction using salting out buffers followed by hydrolysis and peptide characterisation and synthesis for the generation and identification of potential application of bioactive peptides, specifically those related to the Renin-Angiotensin-Aldosterone System (RAAS) [7]. Nine Angiotensin-I-converting enzyme (ACE-I) inhibitory peptides with the sequences FFG, FFFL, PFL, WWK, WCY, FPIL, CPA, FLLA, FEPL and were found to inhibit ACE-I by 96.5% compared to the positive and commercially available drug Captopril^®^ when assayed in vitro at a concentration of 1 mg protein/mL. In addition to this paper, Feeney and colleagues looked at the potential health benefits of glycosylated food proteins, specifically Glycomacropeptide (GMP), which is the terminal end kappa casein and is released from whey during the manufacture of cheese [8]. Using cell assays with HT-29 and Caco-2 cells, they assessed the ability of GMP to prevent adhesion of pathogenic *Escherichia coli* to the cell epithelium and concluded that GMP could be used as a gut health ingredient to protect the gut lining from damage [8]. In addition, Vijayakumar and Muriana discuss the potential use of the bioactive peptides produced from bacteria known as bacteriocins as a food protection agent [9]. Bacteriocins may be defined as ribosomal synthesized antimicrobial peptides produced by bacteria, which can kill or inhibit bacterial strains closely-related or non-related to produced bacteria, but will not harm the bacteria themselves by specific immunity proteins [10].

Novel proteins from seaweeds and macroalgae are reviewed by Bleakley and colleagues [11] and this review includes extraction and characterisation methods for these novel proteins including filtration and mass spectrometry methods. It also details the challenges faced in the development of this new protein resource including access, processing, characterisation, formulation and market challenges including legislation and consumer challenges such as sensory and taste. Total utilisation of by-products or co-products from food processing is important, not just in terms of the bio-economy, but it can also reduce environmental and economic costs for the processors while at the same time developing new products and markets [12]. In this special issue, Ofori and Hsieh examined the use of a monoclonal antibody to detect an antigenic protein in the red blood cells of porcine blood [13]. This development has potential for use by industry to protect consumers from eating porcine blood in food products and could be used for the vegan, vegetarian, Halal and Kosher product development markets. Finally, the review by Henchion and colleagues details potential future protein markets and consumption trends and provides an overview of the positive and negative attributes associated with meat, vegetable, marine and novel protein sources including insect, algal and others [14].
